# Clinical and molecular surveillance of drug resistant vivax malaria in Myanmar (2009–2016)

**DOI:** 10.1186/s12936-017-1770-7

**Published:** 2017-03-16

**Authors:** Myat Htut Nyunt, Jin-Hee Han, Bo Wang, Khin Myo Aye, Kyin Hla Aye, Seong-Kyun Lee, Ye Htut, Myat Phone Kyaw, Kay Thwe Han, Eun-Taek Han

**Affiliations:** 1 Department of Medical Environmental Biology and Tropical Medicine, School of Medicine, Kangwon National University, Chuncheon, Republic of Korea; 2 Department of Medical Research, Yangon, Myanmar; 3 Department of Clinical Laboratory, The First Affiliated Hospital of Anhui Medical University, Anhui, China

**Keywords:** Malaria, Drug resistance, *Plasmodium vivax*, Myanmar, Molecular surveillance

## Abstract

**Background:**

One of the major challenges for control and elimination of malaria is ongoing spread and emergence of drug resistance. While epidemiology and surveillance of the drug resistance in falciparum malaria is being explored globally, there are few studies on drug resistance vivax malaria.

**Methods:**

To assess the spread of drug-resistant vivax malaria in Myanmar, a multisite, prospective, longitudinal study with retrospective analysis of previous therapeutic efficacy studies, was conducted. A total of 906 from nine study sites were included in retrospective analysis and 208 from three study sites in prospective study. Uncomplicated vivax mono-infected patients were recruited and monitored with longitudinal follow-up until day 28 after treatment with chloroquine. Amplification and sequence analysis of molecular markers, such as mutations in *pvcrt*-*O*, *pvmdr1*, *pvdhps* and *pvdhfr*, were done in day-0 samples in prospective study.

**Results:**

Clinical failure cases were found only in Kawthaung, southern Myanmar and western Myanmar sites within 2009–2016. Chloroquine resistance markers, *pvcrt*-*O* ‘AAG’ insertion and *pvmdr1* mutation (Y976F) showed higher mutant rate in southern and central Myanmar than western site: 66.7, 72.7 vs 48.3% and 26.7, 17.0 vs 1.7%, respectively. A similar pattern of significantly higher mutant rate of antifolate resistance markers, *pvdhps* (S382A, K512M, A553G) and *pvdhfr* (F57L/I, S58R, T61M, S117T/N) were noted.

**Conclusions:**

Although clinical failure rate was low, widespread distribution of chloroquine and antifolate resistance molecular makers alert to the emergence and spread of drug resistance vivax malaria in Myanmar. Proper strategy and action plan to eliminate and contain the resistant strain strengthened together with clinical and molecular surveillance on drug resistance vivax is recommended.

**Electronic supplementary material:**

The online version of this article (doi:10.1186/s12936-017-1770-7) contains supplementary material, which is available to authorized users.

## Background

*Plasmodium vivax* is the most globally widespread malaria parasite, causing significant high public health issues in many countries. World Health Organization (WHO) estimated 13.8 million vivax cases globally in 2015. Although vivax was believed to be a benign infection, death related to severe vivax malaria was estimated around 1400–14900 cases in 2015 globally [1].

A decreasing trend in malaria promises the possibility of malaria elimination and many endemic countries are moving forward to malaria elimination [1]. One of the major challenges to achieve the elimination goal is drug-resistant falciparum and vivax infection. Unlike falciparum, drug-resistant vivax is difficult to detect, confirm and monitor because of the nature of vivax malaria, such as the presence of hypnotize stage, low level parasitaemia, asymptomatic carriers, and lack of long-term in vitro testing [2, 3].

In the era of pre-elimination, relatively increased prevalence on vivax was observed [1]. In Myanmar, vivax malaria was the second most common malaria species composed of approximately 30 percent of all malaria cases until 2014 [1]. Afterward, the relative prevalence of the vivax has been increasing. Chloroquine (Chloroquine Phosphate Tablet BP, Remedica Ltd-Cyprus) 25 mg/kg for 3 days followed by primaquine (Remedica Ltd-Cyprus) 0.25 mg/kg for 14 days is the recommended treatment for vivax malaria in Myanmar while ACT (artemisinin-based combinational therapy) has been using to treat falciparum malaria since 2003. Although artemisinin resistance falciparum malaria was confirmed by clinically and molecular approaches, very few document on drug resistance vivax malaria was reported in Myanmar.

Chloroquine-resistant vivax malaria was first reported in Papua New Guinea in 1989 [2]. Chloroquine-resistant vivax has been confirmed in ten countries, including Myanmar [1], and treatment failure within day 28 or chemoprophylaxis failure with chloroquine was reported in 21 countries [3]. Unfortunately, there are no accepted and validated molecular markers for chloroquine or other anti-malarial for resistant vivax malaria. However, potential molecular makers for chloroquine-resistant vivax, such as mutations in *pvcrt*-*O* (*P. vivax* chloroquine resistance transporter-O) and *pvmdr1* (*P. vivax* multidrug resistance protein 1) and antifolate resistant vivax such as *pvdhps* (*P. vivax* dihydropteroate synthetase) and *pvdhfr* (*P. vivax* dihydrofolate reductase) were used for molecular detection to estimate the underlying drug resistance. As there is no documented study on molecular markers analysis on vivax malaria, clinical and molecular markers analysis was conducted in multi-sentinel sites study in Myanmar.

## Methods

### Study site and participants

**Fig. 1:**
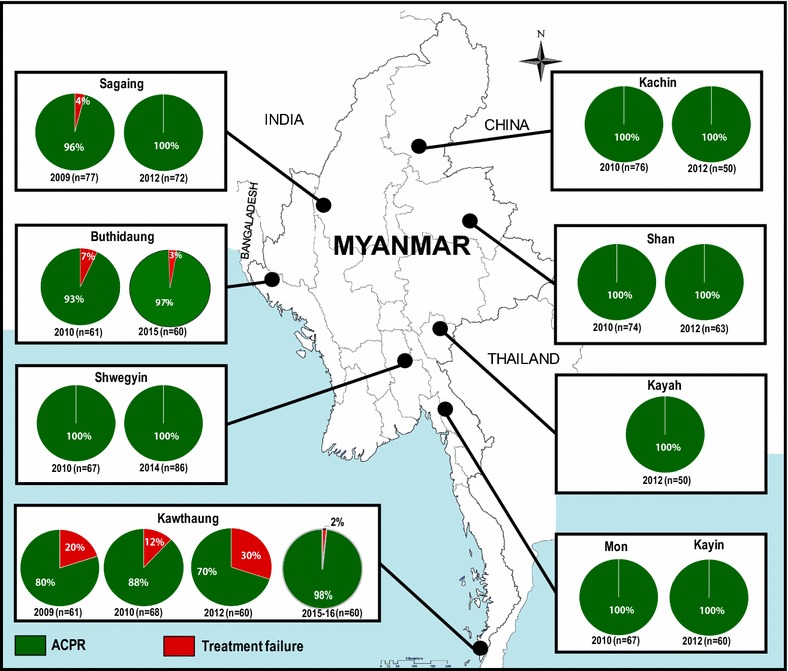
Retrospective and prospective analysis of the clinical surveillance of drug resistance in vivax malaria in Myanmar (2009–2016). Retrospective analysis was conducted in 906 vivax infected cases in nine sentinel sites. Prospective study with molecular surveillance was conducted in Buthidaung (western Myanmar in 2015), Shwegyin (central Myanmar in 2014) and Kawthaung (southern Myanmar in 2015–2016). Clinical failure cases were found only at southern and western Myanmar

The study included the retrospective analysis of previously conducted therapeutic efficacy studies (TES) of chloroquine in uncomplicated vivax malaria in Myanmar and prospective multi-site, longitudinal study by clinical and molecular markers analysis. From 2009 to 2012, TES on vivax malaria was conducted in nine sentinel sites (Fig. 1) which covered most of the malaria-endemic areas in Myanmar. In 2012, Shwegyin, in the southern part of central Myanmar, was selected for TES of chloroquine as this study site has a high burden of both falciparum and vivax malaria among migrant goldmine workers. In 2015–2016, TES of chloroquine on vivax malaria was conducted in Buthidaung, in the western border area and Kawthaung, southern Myanmar.

### Procedure

All procedures on case selection, recruitment and follow-up were carried out according to the standardized protocol recommended by WHO [4]. Briefly, uncomplicated vivax mono-infected patients over 6 years old with fever or history of fever within previous 48 h, were recruited and treated with chloroquine standard dose calculated by body weight, followed by observation with follow-up schedule, i.e., day 3, 7, 21 and 28 after treatment. Clinical and parasitological assessment was done on each follow-up day. All anti-malarials used in this study were provided by the national malaria control programme.

Blood samples were taken on day 0 and all follow-up days for microscopic examination and molecular analysis. Blood film examination on peripheral blood smear was carried out after 3% Giemsa stain for 45 min. Blood film examination was done as described [4] and calculated as parasite count per µL of blood. For molecular analysis, finger-prick blood samples were collected on filter paper (Whatman©), dried and stored in plastic bags with desiccant until analysis. Molecular markers analysis was conducted only in prospective study sites such as Kawthaung, Shwegyin and Buthidaung.

### Molecular analysis

All day-0 samples were subjected to analysis for distribution and spread of chloroquine and antifolate drug resistance markers. Filter papers were prepared and extracted for parasite genomic DNA using QIAamp DNA Blood Mini Kit (QIAGEN) according to manufacturer’s instruction. The target genes were amplified by using the specific pair of primers (Table 1). In this study, chloroquine resistance marker, ‘AAG’ insert in *pvcrt*-*O*; multi-drug resistance marker, mutations in *pvmdr1*; antifolate resistance makers, mutations in *pvdhps* and *pvdhfr* were amplified and analysed.

**Table 1:** Pairs of primers used to amplify the target genes

Target gene	Primer name	Primer sequences (5’→3’)
*pvcrt*-*O*	Pvcrto_F	TCC TTG CCG CTG ATT CTA CG
	Pvcrto_R	GGT AAC GTT CAT CGG GGG TT
*pvmdr1*	Pvmdr1_F3	GGA TAG TCA TGC CCC AGG ATT G
	Pvmdr1_R3	CAT CAA CTT CCC GGC GTA GC
*pvdhfr*	Pvdhfr_F1	ATG GAG GAC CTT TCA GAT GTA TT
	Pvdhfr_R1	CCA CCT TGC TGT AAA CCA AAA AGT CCA GAG
*pvdhps*	Pvdhps_D	GGT TTA TTT GTC GAT CCT GTG
	Pvdhps_B	GAG ATT ACC CTA AGG TTG ATG TAT C

PCR reactions were performed in a reaction mixture that contained 0.25 mM of each dNTP, 10 mM Tris–HCl (pH 9.0), 30 mM KCl, 1.5 mM MgCl_2_, 1.0 units of *Taq* polymerase (Accupower^©^ premix, Bioneer, Seoul, Korea), 0.02 µM primers, and 2 µL of genomic DNA.

For target gene amplifications, initial denaturation at 95 °C for 10 min was followed by 35 cycles of 95 °C for 30 s, 58 °C (*pvdhps*) or 60 °C (*pvcrt*-*O*) or 62 °C (*pvdhps* and *pvdhfr*) for 45 s, 72 °C for 1 min, and a final extension of 72 °C for 10 min. Amplified products were checked by 1% agarose gel electrophoresis stained with Red safe^©^ (iNtRON, Seongnam, Republic of Korea). The PCR clean-up was proceeded by MEGAquick-spin DNA fragment purification Kit (iNtRON, Republic of Korea) and sequencing. The nucleotide and amino acid sequences were aligned and analysed by Lasergene^®^ software (DNASTAR, Madison, WI, USA) using the reference strain of Sal-1 retrieved from Plasmodium data base [5]. The nucleotide sequences were submitted to GenBank under accession numbers KX000945–KX000959.

### Statistical analysis

For clinical data, efficacy outcomes were classified as adequate clinical and parasitological response (ACPR) for successful cure cases until day 28 after treatment or treatment failure (TF) that may be early treatment failure (ETF): failure until day 3 after treatment; or late treatment failure (LTF): failure within days 3 to 28. Data were counter-checked and analysed by MS Excel and SPSS (Version 22.0. IBM Corp., Armonk, NY, USA). Pearson’s Chi squared test or Fisher’s exact test was used to determine with a *P* value of <0.05 accepted significant. Mann–Whitney test was calculated for non-parametric analysis on non-normal distribution. Individual as well as co-occurrence of mutations were analysed and compared among different sentinel sites.

### Ethical consideration

Participation in this study was entirely voluntary. Written consent was taken from all participants. This project obtained ethical clearance from the ethical committee of the Department of Medical Research, Republic of the Union of Myanmar (Approval No-52/Ethics, 2012 and 31/Ethics, 2015) and institutional ethical committee of the Kangwon National University, Republic of Korea (KWNUIRB-2016-04-005).

## Results

### Retrospective analysis of TES

During 2009–2012, TES of chloroquine on 906 uncomplicated vivax malaria infected cases was conducted in nine sentinel sites in Myanmar (Fig. 1). LTF cases, according to the WHO definition [4], were reported in Kawthaung (southern Myanmar), Sagaing and Buthidaung (western Myanmar) sites only while there was no treatment failure cases until day 28 follow-up in remaining study sites. The highest failure rate was noted in Kawthaung in 2012 (18/60, 30%). Kawthaung and Buthidaung site showed failure cases in every study period. There was no early treatment failure case in all study sites.

### Prospective longitudinal study

Kawthaung (southern Myanmar), Shwegyin (central Myanmar) and Buthidaung (western Myanmar) were selected for prospective longitudinal study after treatment with chloroquine regimens. A total of 208 uncomplicated vivax cases were included in this study with mean age of 24.2 (±9.03) years and geometric mean of the parasite density of 4160 with 95% CI (3567–4851) (Table 2). There was no clinical failure case in Shwegyin, but one LTF case in Kawthaung at day 28 (1/60, 1.7%) and two in Buthidaung at days 21 and 28 (2/60, 3.3%).

**Table 2:** Basic characteristics of the study participants

Characteristics	All site (n = 208)	Kawthaung (n = 60)	Shwegyin (n = 88)	Buthidaung (n = 60)	*P* value
Mean age (SD)	24.16 (9.03)	26.27 (10.72)	22.59 (7.99)	24.35 (8.30)	0.091
M:F	1.6:1:0	9.0:1.0	4.5:1.0	60.0:0.0	0.002
Parasite count (p/μL) geometric mean (95% CI)	4160 (3567–4851)	3325 (2404–4597)	4795 (3866–5948)	4225 (3140–5685)	0.520
ACPR (n, %)	205, 98.6	59, 98.3	88, 100.0	58, 96.7	0.258

*SD* standard deviation, *M* male, *F* female, *CI* Confident interval, *ACPR* adequate clinical and parasitological response

### Molecular marker analysis

**Fig. 2:**
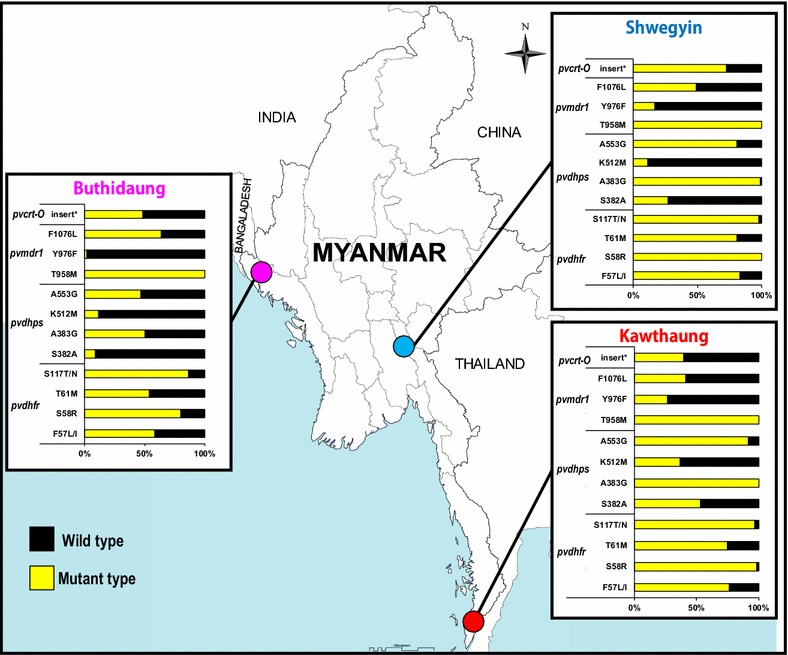
Molecular marker analysis of drug resistance in vivax malaria in three sentinel sites in Myanmar (2014–2016). A total of 208 from three sentinel sites were included for molecular marker analysis. K10 (AAG) insert of *pvcrt*-*O* gene, and mutations of *pvmdr1*, *pvdhps* and *pvdhfr* were shown. Among three sites, the prevalence of all markers except F1076L of *pvmdr1* were fewest in Buthidaung site

All day-0 samples collected from three sentinel sites were analysed for molecular markers (Fig. 2; Table 3). More than half of the samples (133/204, 63.9%) showed K10 ‘AAG’ insertion in chloroquine resistance transporter gene, *pvcrt*-*O*. Among them, Shwegyin showed the highest mutant rate (64/88, 72.7%) followed by Kawthaung (40/60, 66.7%) and Buthidaung (29/60, 48.3%). Similarly, *pvmdr1* (Y976F) was found in Kawthaung, Shwegyin and Buthidaung as 16/60, 26.7%; 15/88, 17.0% and 1/60, 1.7%, respectively. Interestingly, significant highest mutant rate of F1076L was observed in Buthidaung (38/60, 63.3%) (p = 0.067). To estimate the chloroquine resistance status, *pvcrt*-*O* (K10 insertion) and *pvmdr1* mutations (Y976F and F1076L) were analysed together. Only K10 insertion was lowest in Buthidaung (29/60, 48.3%) with highest rate of F1076L alone or with K10 insertion together. However, the number of isolates showing K10 insert in *pvcrt*-*O* gene as well as both *pvmdr1* mutations (Y976F and F1076L) was highest in Kawthaung (11/60, 16.7%) followed by Shwegyin (11/88, 12.5%) and no isolate in Buthidaung (Tables 3, 4). Similarly, all mutations of *pvdhps* (S382A, A383G, K512M, A553G) showed the highest in Kawthaung (17/60, 28.3%) followed by Shwegyin (6/88, 6.8%) and Buthidaung (2/60, 3.3%) (Table 3). Moreover, half of the samples showed the wild type *pvdhps* alleles while no wild type in Kawthaung and one only (1/88, 1.1%) in Shwegyin (Table 4).

**Table 3:** Prevalence of the individual single nucleotide polymorphisms (SNPs) in all study sites

Target genes	Kawthaung (n = 60)	Shwegyin (n = 88)	Buthidaung (n = 60)	All site (n = 208)	*P* value
*pvcrt*-*O*					
‘AAG’ insert	40 (66.7)	64 (72.7)	29 (48.3)	133 (63.9)	0.009
*pvmdr1*					
T958M	60 (100.0)	88 (100.0)	60 (100.0)	208 (100.0)	NR
Y976F	16 (26.7)	15 (17.0)	1 (1.7)	32 (15.4)	0.001
F1076L	25 (41.7)	43 (48.9)	38 (63.3)	106 (51.0)	0.067
*pvdhps*					
S382A	32 (53.3)	24 (27.3)	5 (8.3)	61 (29.3)	0.000
A383G	60 (100.0)	87 (98.9)	30 (50.0)	177 (85.1)	0.000
K512M	22 (36.7)	10 (11.4)	7 (11.7)	39 (18.8)	0.000
A553G	55 (91.7)	71 (80.7)	28 (46.7)	154 (74.0)	0.000
*pvdhfr*					
F57L/I	46 (76.7)	73 (83.0)	35 (58.3)	154 (74.0)	0.001
S58R	59 (98.3)	88 (100.0)	48 (80.0)	195 (93.8)	0.000
T61M	45 (75.0)	71 (80.7)	32 (53.3)	148 (71.2)	0.001
S117T/N	58 (96.7)	86 (97.7)	52 (86.6)	196 (94.2)	0.003

Prevalence of SNPs were shown as ‘number (percent)’ of respective site. All P value were calculated by Chi square test with 95% CI

*NR* not relevant to be calculated

**Table 4:** Co-prevalence of single nucleotide polymorphisms (SNPs) in different molecular markers in sentinel sites

Target	Description^a^	No. isolate/total (%)			
		Kawthaung (n = 60)	Shwegyin (n = 88)	Buthidaung (n = 60)	All sites (n = 208)
*pvcrt*-*O*	Wild type	20 (33.3)	24 (27.3)	31 (51.7)	75 (36.1)
	Mutant (AAG insert)	40 (66.7)	64 (72.7)	29 (48.3)	133 (63.9)
*pvmdr1*	Wild type (T, Y, F) (958, 976, 1976)	0 (0.0)	0 (0.0)	0 (0.0)	0 (0.0)
	Single mutant (**M**, Y, F)	35 (58.3)	46 (52.3)	22 (36.7)	103 (49.5)
	Double mutant (**M**, Y, **L/I**)	9 (15.0)	27 (30.7)	37 (61.7)	73 (35.1)
	Triple mutant (**M**, **F**, **L**)	16 (26.7)	15 (17.0)	1 (1.7)	32 (15.4)
*pvdhps*	Wild (S, A, K, A) (382, 383, 512, 553)	0 (0.0)	1 (1.1)	30 (50.0)	31 (14.9)
	Single mutant (S, **G**, K, A)	5 (8.3)	15 (17.0)	2 (3.3)	22 (10.6)
	Double mutant (S, **G**, K, **G**)	18 (30.0)	45 (51.1)	18 (30.0)	81 (38.9)
	Double mutant (**A**, **G**, K, A)	0 (0.0)	1 (1.1)	0 (0.0)	1 (0.5)
	Triple mutant (**A**, **G**, K, **G**)	15 (25.0)	16 (18.2)	3 (5.0)	34 (16.3)
	Triple mutant (S, **G**, **E**, **G**)	0 (0.0)	2 (2.3)	0 (0.0)	2 (1.0)
	Triple mutant (S, **G**, **M**, **G**)	5 (8.3)	1 (1.1)	5 (8.3)	11 (5.3)
	Quadruple mutant (**A**, **G**, **M**, **G**)	17 (28.3)	6 (6.8)	2 (3.3)	25 (12.0)
	Quadruple mutant (**C**, **G**, **E**, **G**)	0 (0.0)	1 (1.1)	0 (0.0)	1 (0.5)
*pvdhfr*	Wild (F, S, T, S) (57, 58, 61, 117)	0 (0.0)	0 (0.0)	5 (8.3)	5 (2.4)
	Single mutant (**L**, S, T, S)	1 (1.7)	0 (0.0)	2 (3.3)	3 (1.4)
	Single mutant (F, **R**, T, S)	1 (1.7)	0 (0.0)	0 (0.0)	1 (0.5)
	Single mutant (F, S, T, **T/N**)	0 (0.0)	0 (0.0)	5 (8.3)	5 (2.4)
	Double mutant (**L**, **R**, T, S)	0 (0.0)	2 (2.3)	1 (1.7)	3 (1.4)
	Double mutant (F, **R**, T, **N**)	13 (21.7)	14 (15.9)	15 (25.0)	42 (20.2)
	Triple mutant (**L**, **R**, T, **T**)	0 (0.0)	1 (1.1)	0 (0.0)	1 (0.5)
	Triple mutant (F, **R**, **M**, **T**)	0 (0.0)	1 (1.1)	0 (0.0)	1 (0.5)
	Quadruple mutant (**L/I**, **R**, **M**, **T/N**)	45 (75.0)	70 (79.5)	32 (53.3)	147 (70.7)

^a^ Numbers in parentheses indicate the amino acid position. Mutant amino acids are shown in bold. All sequences were aligned with Sal-1 (*P. vivax*) reference sequences from Plasmodium data base

Furthermore, mutations in *pvdhfr* gene were analysed and all mutations, F57L/I, S58R, T61M, and S117T/N, showed the highest mutant rate in Shwegyin (70/88, 79.5%) followed by Kawthaung (45/60, 75.0%). Wild type alleles were found only in Buthidaung (5/60, 8.3%). Triple or quadruple mutant alleles of *pvdhps* was observed and accounted for more than 50% of samples in all study sites (Table 4). A combined analysis of all mutations in 208 samples totaled 80 different genotypes (Additional file 1).

## Discussion

This study explores the clinical and molecular pattern of candidate drug resistance markers in Myanmar. Retrospective and prospective analysis on TES of chloroquine on vivax malaria (2009–2016) showed that clinical failures were detected only in southern and western Myanmar. Molecular markers analysis augments additional information on the pattern of drug resistance. In this study, molecular maker analysis indicated widespread distribution of chloroquine, antifolate and multidrug resistance markers, with the highest mutant rate in southern Myanmar.

Surveillance on drug resistance malaria is of great concern for global control and elimination of malaria. As drug-resistant vivax was reported as early as 1980s, its exact global burden is still unknown. Compared to falciparum malaria, drug resistance studies of vivax which focus on epidemiology, drug efficacy and drug resistance mechanism are rare. Drug resistance malaria can be detected by in vivo TES studies, molecular maker analysis, in vitro drug susceptibility testing and drug concentration measurement.

Lack of a standardized culture system limits the usefulness of in vitro susceptibility tests for detection of drug resistance. Although in vivo TES were accepted as a standard method for drug resistance detection, recurrent parasitaemia cases needed to be distinguished between re-infection, recrudescence or relapse. Relapse patterns of vivax malaria also widely differ across geographical regions and no standardized method to exclude relapse or re-infection in TF cases leads to difficulties in interpretation of findings of TES. Currently, clinical surveillance with chloroquine drug level measurement has been accepted to confirm chloroquine-resistant vivax malaria. At least one TF case that showed whole blood concentration of chloroquine plus desethylchloroquine more than 100 ng/mL was observed in ten countries: Brazil, Ethiopia, Indonesia, Malaysia (Borneo), Myanmar, Papua New Guinea, Peru, the Solomon Islands, and Thailand [6].

Although there are no validated molecular markers for drug-resistant vivax malaria, potential candidates were reported. Most of these candidate markers [7–9] were homologues of falciparum drug resistance makers, such as *pvmdr1*, *pvdhp* and *pvdhfr*. K10 insertion (‘AAG’ insert) in first exon at tenth position of *pvcrt*-*O* was also suggested as a chloroquine resistance marker [7, 10]; it was observed in Thailand (56–89%) [7, 10] and Myanmar (46%) [7]. In this study, the highest rate of K10 inset alleles was observed in Shwegyin (central Myanmar) (72.7%) followed by Kawthaung (southern Myanmar) (66.7%) and Buthidaung (western Myanmar) (48.3%) indicating high chloroquine resistance in southern Myanmar, which is similar to the artemisinin resistance status [11]. As the chloroquine is the first line treatment for vivax malaria in Myanmar, K10 insert of *pvcrt*-*O* gene was widely distributed in all three study sites.

Y976F mutation of *pvmdr1* was found to be associated with reduced susceptibility of chloroquine in Thailand and Indonesia [12] but not in Madagascar [13]. Moreover, Y976F of *pvmdr1* gene has been associated with higher susceptibility to artesunate and mefloquine [12]. In this study, Y976F was detected in Kawthaung (26.7%), Shwegyin (17.0%) and Buthidaung (1.7%). Compared to neighbouring countries, Y976F was highest in Cambodia (89%) [14], followed by Thailand (8–25%) [10, 14, 15], China–Myanmar border (3%) [16], and India (0%) [17]. Another mutation of *pvmdr1*, F1096L was observed widely but was suggested to be neutral for drug resistance [18]. In combined analysis of *pvcrt*-*O* and *pvmdr1* mutations, chloroquine resistance markers were widely distributed in all three study sites but with a lower mutant rate in western Myanmar.

Similarly, *pvdhps* and *pvdhfr* showed a significant role in antifolate drug-resistant vivax malaria [19]. F57I/L, S58R, T61M, and S117T/N of *pvdhfr* were found to be associated with pyrimethamine resistance [20] and S382A, S383G, A553G of *pvdhps* were associated with sulfadoxine resistance [21]. *Plasmodium vivax* was supposed to have a certain degree of innate resistance to sulfadoxine and S383G and A553G could be responsible inducers for resistance [22]. In Myanmar, relatively high rates of these mutations were noted in southern and central Myanmar.

Double mutations (S58R and S117T/N) or quadruple mutations (F57L/I, S58R, T61M, and S117T) of *pvdhfr* gene were found in Thailand (96%), Myanmar (71%), Korea (1%), Cambodia (94%), and India (40%) [23, 24]. In this study, overall quadruple mutations was 147/208 (70.7%) and contributed more than half of samples in all three study sites, indicating high pyrimethamine resistance in Myanmar. According to *pvdhps* and *pvdhfr* data, antifolate resistance in vivax infection in Myanmar should not be neglected, although sulfadoxine-pyrimethamine is not the drug of choice for vivax malaria.

Within 2009–2016, treatment regimen for vivax malaria was not changed and trend on clinical efficacy of chloroquine is similar except some fluctuations in Kawthaung study site. It is difficult to rule-out the drug resistance factor resulting in treatment failure in this study sites without molecular analysis in previous years. As the drug resistance alone is not responsible for treatment failure [4], clinical response and prevalence of molecular markers is not similar in this study.

## Conclusions

This study is the first multisite, clinical and molecular surveillance of drug-resistant vivax malaria in Myanmar, exploring the neglected niche of the infection. According to current anti-malarial treatment guidelines in Myanmar, chloroquine is the first-line treatment for vivax malaria. Most of the study sites showed 100% ACPR except in the southern and western Myanmar sites. Wide distribution of chloroquine and antifolate resistance molecular markers revealed the spread of the drug-resistant parasite population in Myanmar. High mutant rates of most of the vivax drug resistance molecular markers in southern and central Myanmar were similar to that of artemisinin resistance falciparum malaria, indicating higher anti-malarial resistance burden of falciparum and vivax in southern Myanmar. An appropriate strategy and action plan to contain or eliminate drug-resistant vivax malaria in Myanmar is recommended.

## Additional file

**Additional file 1.** Genotypes based on the co-prevalence of the drug resistance vivax makers.
